# Transcriptome sequencing and analysis of rubber tree (*Hevea brasiliensis* Muell.) to discover putative genes associated with tapping panel dryness (TPD)

**DOI:** 10.1186/s12864-015-1562-9

**Published:** 2015-05-21

**Authors:** Jin-Ping Liu, Zhi-Qiang Xia, Xiao-Yan Tian, Yi-Jian Li

**Affiliations:** Hainan Key Laboratory for Sustainable Utilization of Tropical Bioresources, College of Agronomy, Hainan University, Haikou, Hainan Province 570228 P. R China; The Institute of Tropical Bioscience and Biotechnology, Chinese Academy of Tropical Agricultural Sciences, Haikou, Hainan Province 571101 P. R China; Service Center of Science and Technology, Rubber Research Institute Chinese Academy of Tropical Agricultural Sciences, Danzhou, Hainan Province 571737 P. R China

**Keywords:** Rubber tree, TPD, Transcriptome, Latex biosynthesis, Jasmonate synthesis

## Abstract

**Background:**

Tapping panel dryness (TPD) involves in the partial or complete cessation of latex flow thus seriously affect latex production in rubber tree (*Hevea brasiliensis*). Numerous studies have been conducted to define its origin and nature, but the molecular nature and mechanism of TPD occurrence remains unknown. This study is committed to *de novo* sequencing and comparative analysis of the transcriptomes of healthy (H) and TPD-affected (T) rubber trees to identify the genes and pathways related to the TPD.

**Results:**

Total raw reads of 34,632,012 and 35,913,020 bp were obtained from H and T library, respectively using Illumina Hiseq 2000 sequencing technology. *De novo* assemblies yielded 141,456 and 169,285 contigs, and 96,070 and 112,243 unigenes from H and T library, respectively.

Among 73597 genes, 22577 genes were identified as differential expressed genes between H and T library via comparative transcript profiling. A majority of genes involved in natural rubber biosynthesis and jasmonate synthesis with most potential relevance in TPD occurrence were found to be differentially expressed.

**Conclusions:**

In TPD-affected trees, the expression of most genes related to the latex biosynthesis and jasmonate synthesis was severely inhibited and is probably the direct cause of the TPD. These new *de novo* transcriptome data sets provide a significant resource for the discovery of genes related to TPD and improve our understanding of the occurrence and maintainace of TPD.

**Electronic supplementary material:**

The online version of this article (doi:10.1186/s12864-015-1562-9) contains supplementary material, which is available to authorized users.

## Background

Rubber tree (*Hevea brasiliensis* Muell.) is a perennial plant of the Euphorbiaceae family and is commercially cultivated in the tropical areas worldwide (particularly in the Southeast Asia) for the production of natural rubber (cis-1,4-polyisoprene) which is an strategic raw material for over 40,000 products [1,2]. The increased global demand for rubber significantly prompted the cultivation of rubber trees. But rubber production has been facing a serious menace of tapping panel dryness (TPD). Annual rubber production loss from TPD accounted for 15-20% with an incidence of 12-50% [3] and no effective treatments has been developed for it [4]. Latex, a rubber containing cytoplasm component of the laticifers, is harvested by periodic cutting or tapping of the bark and TPD symptom is the cessation of latex flow and reduction of the tapping stands [1]. Additionally, other symptoms including browning, thickening or flaking of the bark can occur.

There have been many prolific attempts to understand the nature and molecular mechanisms of TPD. It was initially hypothesized that TPD might be caused by pathogens [5–7], but no further evidence confirmed it [8–11]. Many researchers proposed that TPD is a physiological anomaly resulted from wounding stress and ethylene overstimulation [12–16]. During the development of TPD, many physiological and biochemical changes were observed to occur including decrease of sucrose and dry matter and increase of inorganic phosphorus in the latex [17–19], increase of activities of RNase and proteinase [20–22] and decrease of the content of protein, nucleic acid, thiols, ascorbic acid, the levels of variable peroxidase and superoxide dismutase isozyme (SOD) [23,24], and the impaired rubber synthesis [25]. Chrestin et al. [26] and Chrestin [12] put forward that uncompensated oxidative stress within the latex cells might be associated with membrane destabilization that ultimately resulted in bursting of the lutoids and consecutive in situ latex coagulation.

Several protein markers linked to TPD have been identified [27–30], but their functions in TPD onset are unclear. A few genes associated with TPD also have been isolated. Chen et al. [3] cloned and characterized a transcription factor *HbMyb1* with mRNA differential display reverse transcriptase polymerase chain reaction (DDRT-PCR), implying that it may play a role in apoptosis. Venkatachalam et al. [31] using DDRT-PCR also identified *HbTOM20* gene (Hevea brasiliensis Translocase of the Outer Mitochondrial Membrane), suggesting that it may involve in alteration of mitochondrial metabolism. Venkatachalam et al. [32] identified a thymidine kinase gene (*HbTK*) related to TPD by random amplified polymorphic DNA screening. Moreover, Venkatachalam et al. [33] and Li et al. [4] studied the expression patterns of the TPD responsive genes using suppression subtractive hybridization (SSH) method and they found that genes associated with stress/defense response preferentially expressed in the fresh latex samples from rubber tree with the onset of TPD syndrome. Qin et al. [34] identified the genes associated with TPD by oligonucleotide microarrays hybridized with the latex from TPD and healthy rubber trees. Although these approaches are helpful to elucidate the onset of TPD, but they still leave many gaps in the knowledge and understanding of the nature and mechanism of TPD.

Recently RNA sequencing (RNA-Seq), referring to the whole-transcriptome shotgun sequencing of fragmented mRNA or cDNA, has emerged as a powerful tool for profiling expressed genes in plants and other organisms [35–41]. RNA-Seq has obvious advantages over existing methodologies such as enabling large-scale functional assignments of genes, more thorough qualitative and quantitative analysis of gene expression, more sensitive and accurate profiling of the eukaryotic transcriptome including non-model organisms [42–45].

In the rubber tree, several reports about the transcriptional profile of different organs and tissues were published [46–52], but the next-generation sequencing-based transcriptome analysis of rubber trees affected by TPD have not been conducted. In order to improve our understanding of the molecular nature and mechanism of TPD, we performed the first comparative transcriptome sequencing and analysis of the barks of healthy rubber trees and the ones affected by TPD using the Illumina RNA-Seq method. Further, in this study we have attempted to identify and characterize the differentially expressed genes involved in the JA synthesis, isopentenyl pyrophosphate (IPP) and transcriptional regulation.

## Results

### Sequencing, de novo assembly and functional annotation

**Figure 1:**
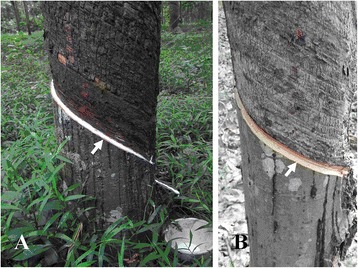
A healthy (H) rubber tree with normal latex flow (indicated by arrow) **(A)** and a TPD-affected (T) rubber tree which tapping panel become completely dry (indicated by arrow) **(B)**.

To identify the genes associated with TPD, two sequencing cDNA libraries were constructed: H library from bark tissue in healthy rubber trees with normal latex flow (Figure 1A) and T library from bark tissue in TPD-affected rubber trees which tapping panel become completely dry (Figure 1B). By sequencing on the platform of Illumina Hiseq 2000, total raw reads of 34,632,012 and 35,913,020 bp were generated from H and T library, respectively. After filtering and removing of reads containing adapter sequences and unknown nucleotides (larger than 5%), and low quality reads, total clean reads of 31,162,830 and 32,330,974 bp were obtained with 4,674,424,500 and 4,849,646,100 nucleotides, respectively. Then, these clean reads were de novo assembly into 141,456 and 169,285 contigs, and further into 96,070 and 112,243 unigenes with a mean length of 637 and 609 bp, a N50 length of 1250 and 1213 bp, a total length of 96,070 and 112,243 bp, respectively. All clean reads were uploaded and deposited in the NCBI Sequence Read Archive (SRA) under the accession numbers of SRR1611790 (for H) and SRR1611792 (for T). The sequencing datasets were also submitted to the Gene Expression Omnibus (GEO) and assigned the accession number of GSE67214.

**Figure 2:**
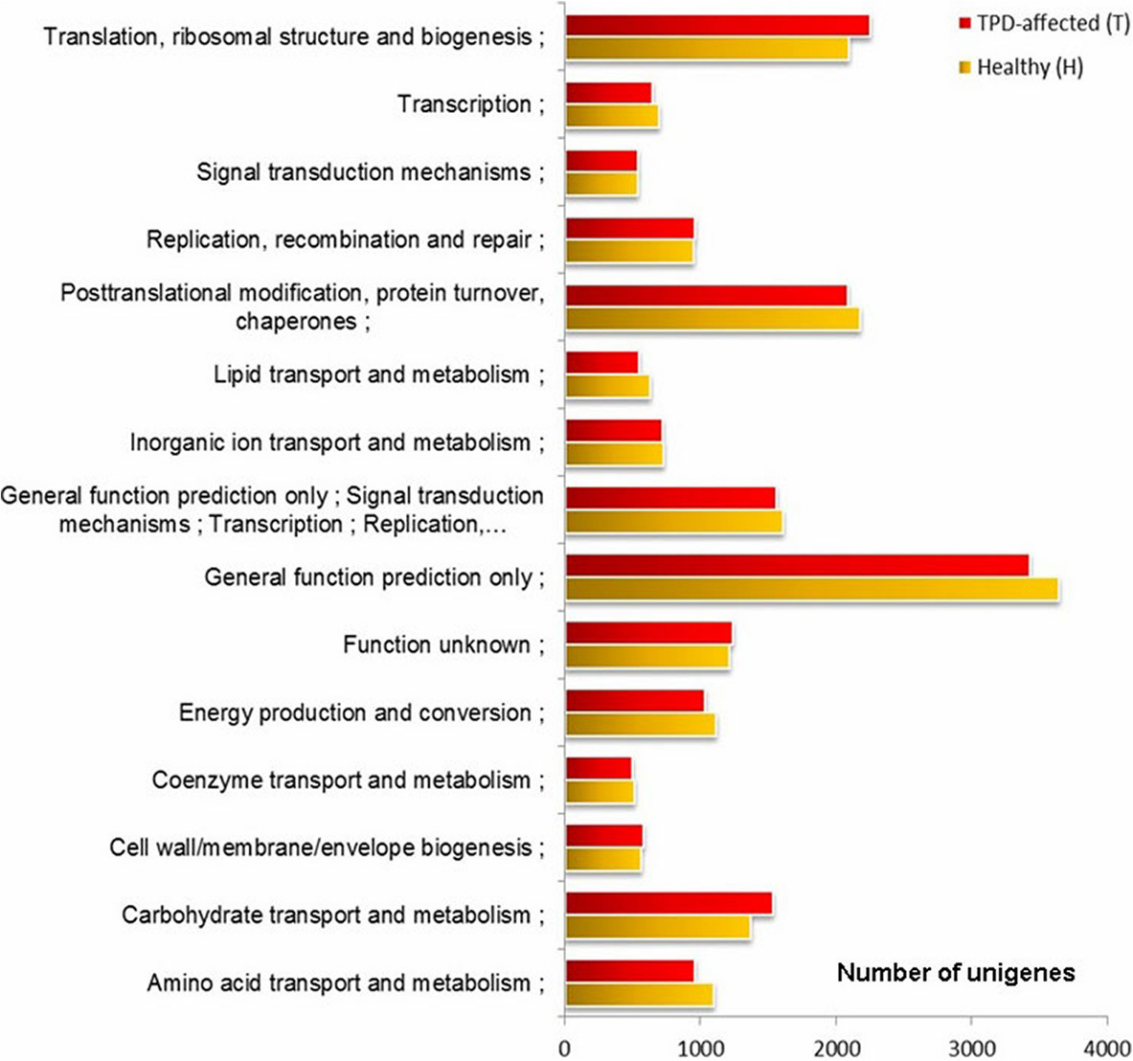
COG functional distribution of the transcriptomes of healthy (H) and TPD-affected (T) rubber tree barks.

For function annotation analysis, 68621, 63025, 44183, 41042, 28238 and 53709 unigenes were annotated to the NR, NT, Swiss-Prot, KEGG, COG and GO database, respectively, with the total annotation unigenes of 73597. The unigenes were assigned to three main categories (the biological process, the cellular component and the molecular function) of the GO classification. COG function classification for H and T is shown in Figure 2. Among the 15 COG categories, general function prediction only was the most highly represented group with 3640 and 3424 genes in H and T libraries respectively, followed by the cluster for translation, ribosomal structure and biogenesis with 2090 and 2253 genes, and the cluster for posttranslational modification, protein turnover, chaperones with 2177 and 2087 genes in H and T libraries respectively. Coenzyme transport and metabolism COG group contains the fewest genes (only 511 and 498 genes in H and T libraries respectively). 41042 unigenes were assigned to 125 KEGG pathways. Of these, assignment to the metabolic pathways made up the majority (9916, 24.16%), followed by biosynthesis of secondary metabolites (4642, 11.31%), plant-pathogen interaction (2497, 6.08% ), ribosome (2044, 4.98%) and plant hormone signal transduction (1903, 4.64%). A detailed list of Gene lengths, Nr annotations, Nt annotations, Swiss-Prot annotations, COG function descriptions, KEGG genes, GO biological processes and GO molecular functions in H and T Transcriptomes was given in Additional file 1.

### Differentially expressed genes (DEGs) in the healthy and TPD affected trees

Unigene expression difference analysis was conducted with the total number of unigenes of 107020 (73597 unigenes with annotations). 22577 annotated DEGs and 29702 DEGs with no function and no hit were obtained between the healthy and TPD affected trees using FDR ≤ 0.001 and |log2Ratio| ≥ 1 as a threshold to determine the statistical significance of gene expreesion (Figure 2). Of all annotated DEGs, 12178 were up-regulated and 10399 were down-regulated.

**Figure 3:**
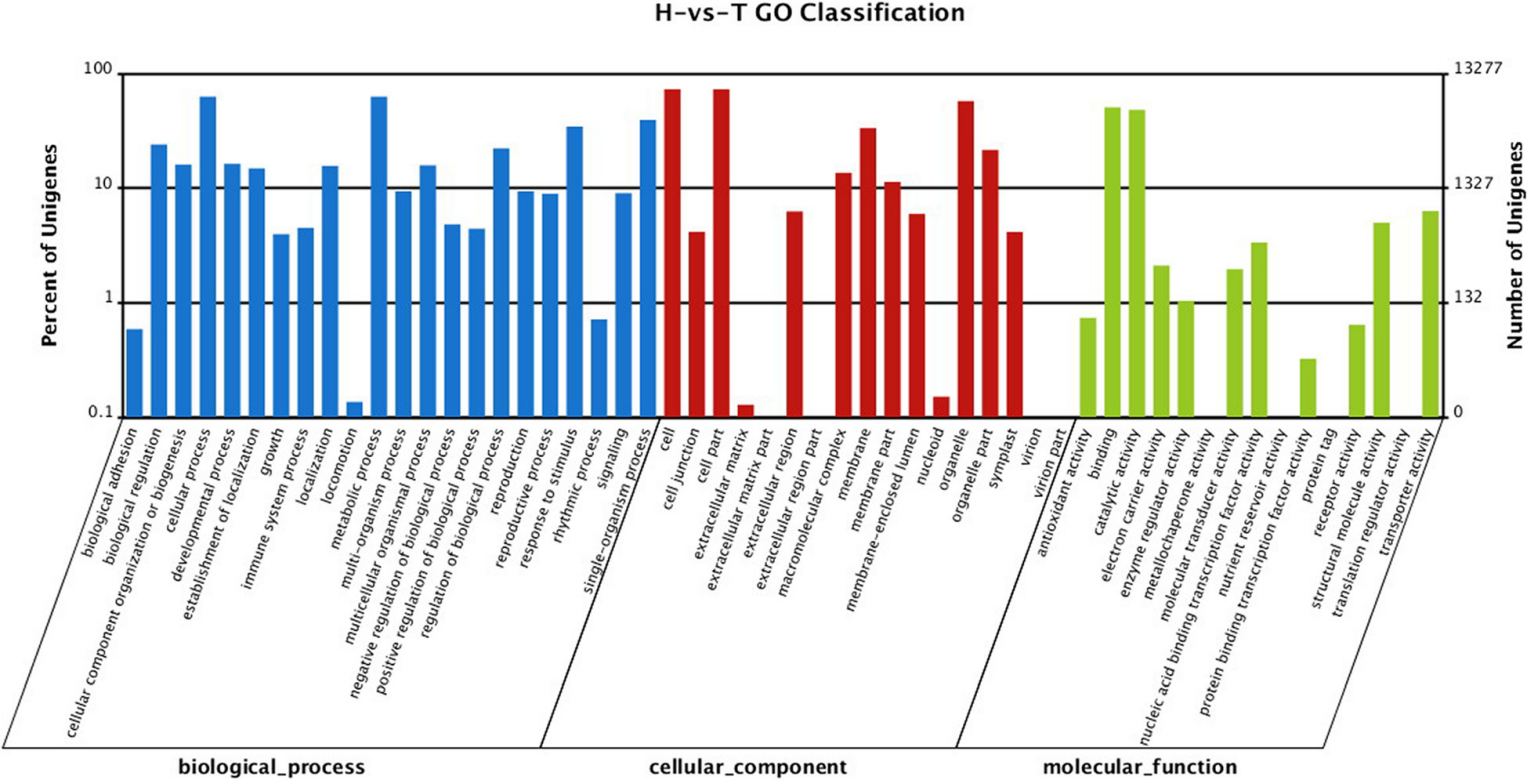
Ontology classification of DEGs in the TPD-affected (T) bark compared to the healthy (H) bark of rubber tree. “H-vs-T” means H is control and T is treated. Distribution of expressed genes in H compared to H with the categories of biological process, cellular components and molecular function. The Y-axis on the right side indicates the percent of genes in a category, and the y-axis on the left means the number of genes.

**Figure 4:**
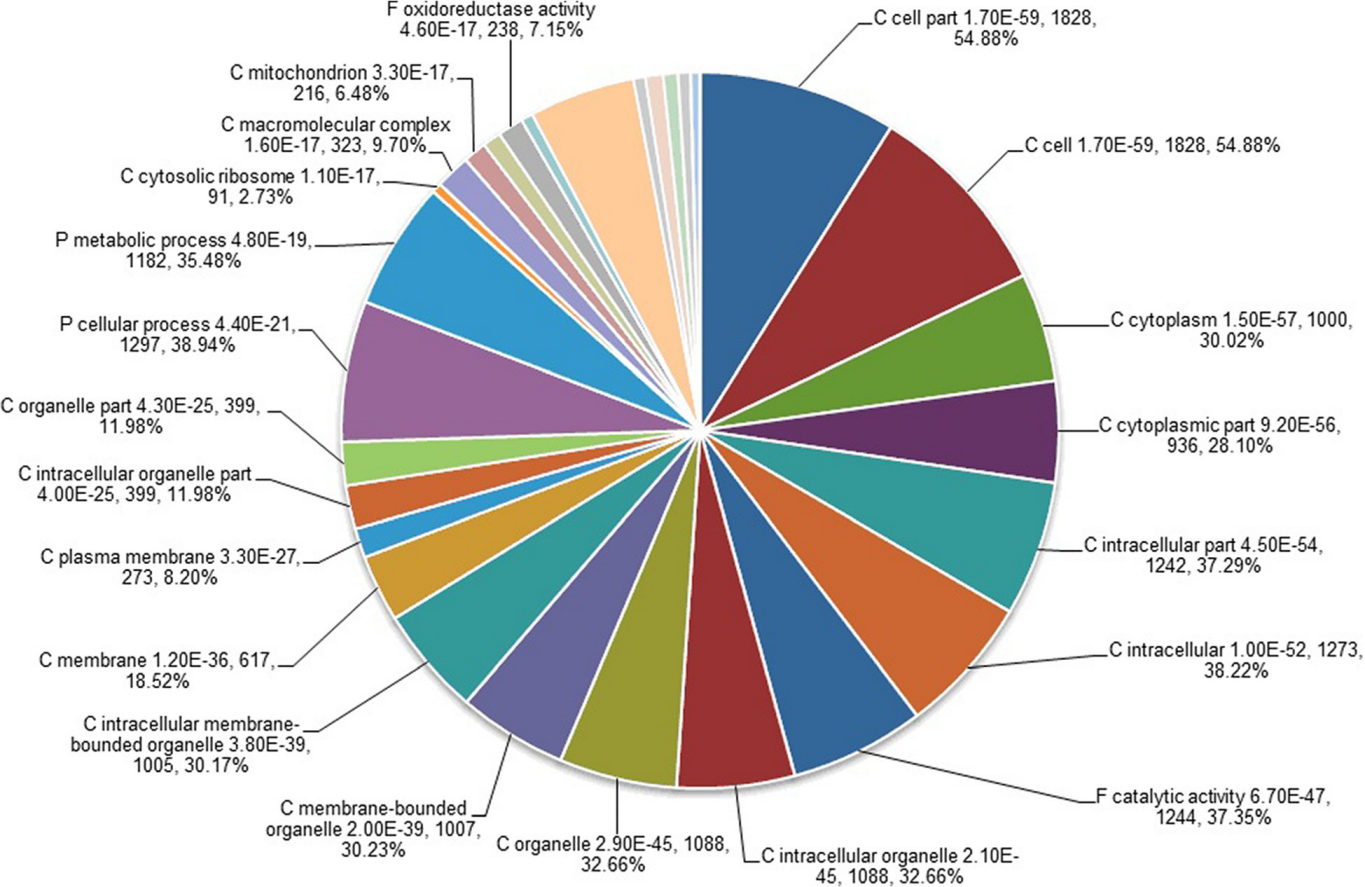
Gene ontology annotation of down-regulated genes in the TPD-affected (T) bark compared to the healthy (H) bark of rubber tree. P: the biological process; C: the cellular component; F: the molecular function. The P value, the number of genes and the percentage of genes in the corresponding categories were given after the name of subcategories.

**Figure 5:**
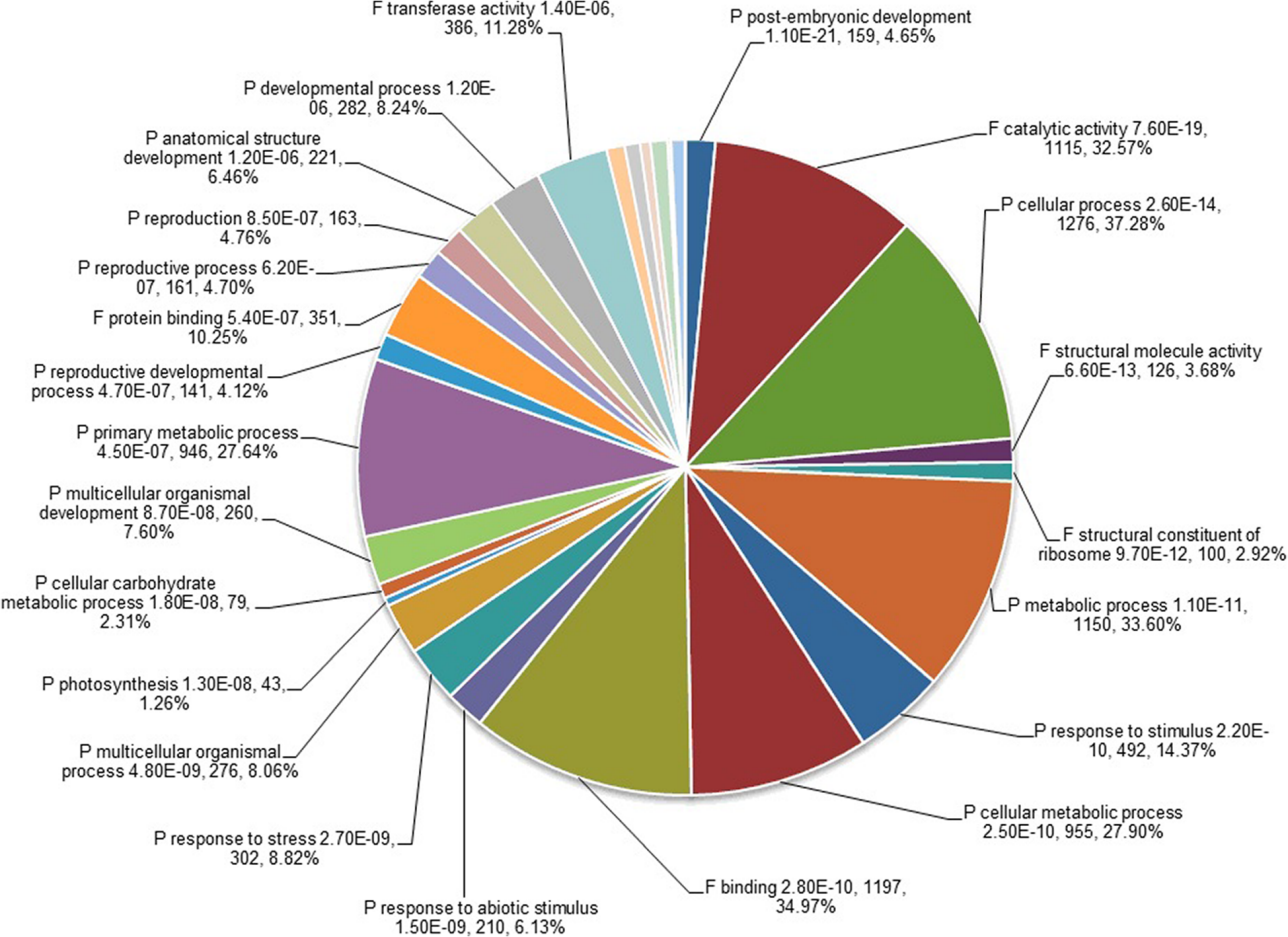
Gene ontology annotation of up-regulated genes in the TPD-affected (T) bark compared to the healthy (H) bark of rubber tree. P: the biological process; C: the cellular component; F: the molecular function. The P value, the number of genes and the percentage of genes in the corresponding categories were given after the name of subcategories.

GO classification system was used to classify the possible functions of the DEGs. A total of 22577 DEGs were assigned to three main categories: the biological process, the cellular component and the molecular function (Figure 3). A high percentage of DEGs in the biological process category fell under “cellular process” and “metabolic process”. “cell” and “cell part” subcategories dominated in the cellular component category, and DEGs related to “binding” and “catalytic activity” were heavily represented the molecular function category. Of 10399 down-regulated genes in T compared to H, “cell” (1828 DEGs, 54.88%) and “cell part” (1828 DEGs, 54.88%), “cellular process” (1297 DEGs, 38.94%) and “metabolic process” (1182 DEGs, 35.48%), and “catalytic activity” (1244 DEGs, 37.35%) were major subcategories in the categories of the cellular component (C), the biological process (P) and the molecular function (F), respectively (Figure 4). Of the up-regulated DEGs in T compared to H, the largest proportion was represented by “cellular process” (1276 DEGs, 37.28%) under the biological process (P) and “binding” (1197 DEGs, 34.97%) under the molecular function (F), followed by “metabolic process” (1150 DEGs, 33.60%) under the biological process (P) and “catalytic activity” (1115 DEGs, 32.57%) under the molecular function (F) (Figure 5).

Of the 2660 DEGs with differences greater than 15-fold, 1206 unigenes were present at higher levels in the healthy trees than the TPD affected trees, and 1454 in the TPD affected trees than the healthy trees. The DEGs exhibiting the highest down-regulated expression in T compared to H were the genes coding 6-phosphogluconolactonase (with a log_2_ ratio (T/H) of −11.347), chain A of anomalous substructure of hydroxynitrile lyase (with a log_2_ ratio (T/H) of −11.0766), uncharacterized protein LOC101298879 (with a log_2_ ratio (T/H) of −10.7788) and osmotin precursor (with a log_2_ ratio (T/H) of −10.6413). The genes coding arginine N-methyltransferase (with a log_2_ ratio (T/H) of 9.655192) and Dip1-associated protein C (with a log_2_ ratio (T/H) of 9.438686) were the top up-regulated DEGs in T compared to H.

### Differentially expression of putative genes related to TPD

In view of the facts that the natural rubber (consists mainly of poly-cis-isoprene) synthesis is involved in isoprenoid metabolism, and TPD syndrome is often an abrupt and complete shut down of the latex biosynthesis machinery, we focused on pathway analysis of IPP metabolism, volatile mevalonic pathway and jasmonate synthesis.

### DEGs involved in isoprenoid biosynthesis

**Figure 6:**
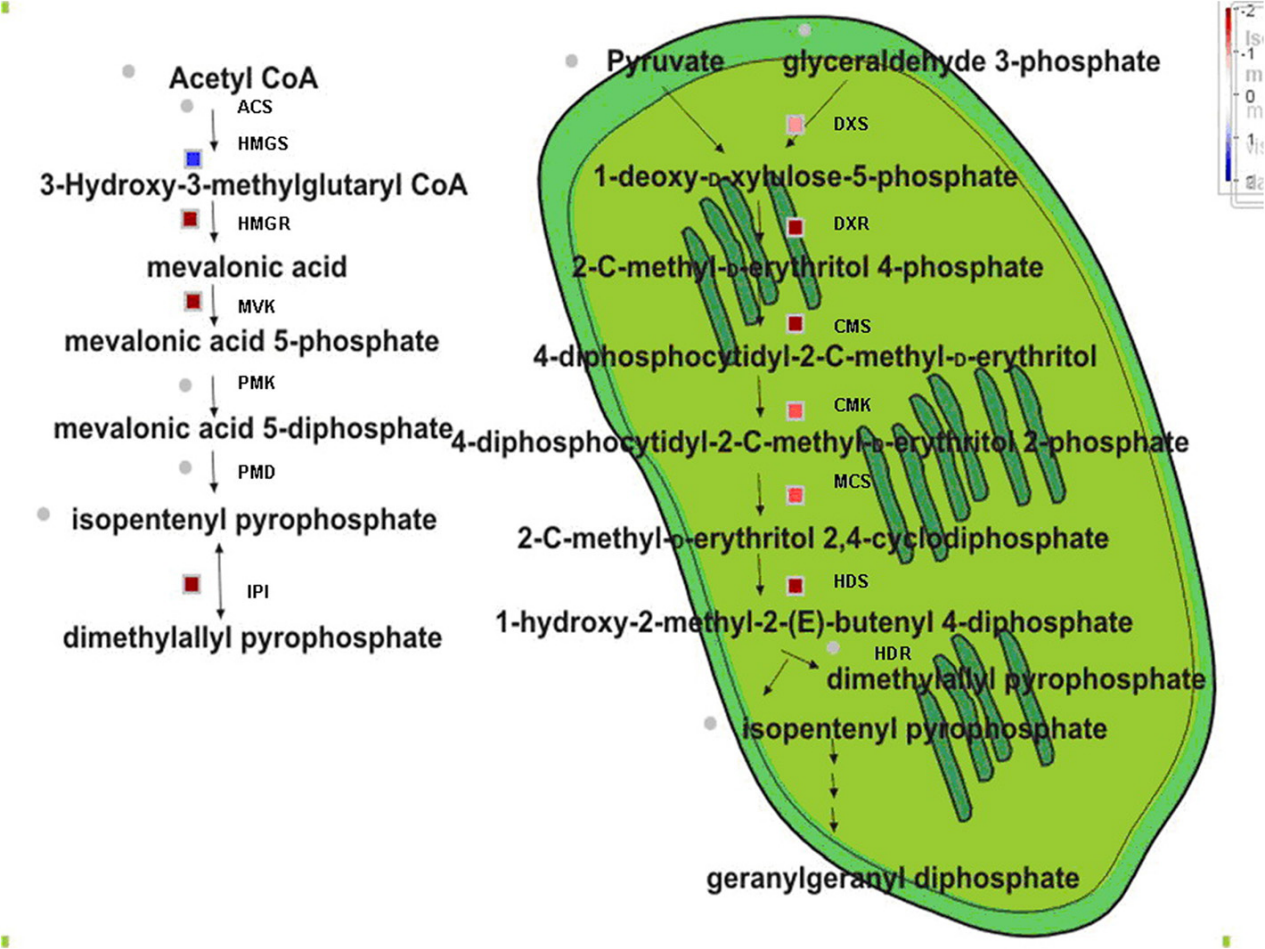
Differential expression of transcripts involved in MVA pathway (left) and MEP pathway (right) across the transcriptome of the TPD affected rubber trees. Color bar represents the levels of transcript abundance. The depth of the red and the blue indicates the levels of the down-regulated expression and the up-regulated expression of the genes, respectively.

Based on the KEGG pathway enrichment analysis, four and six DEGs that encode exzymes involved in mevalonic acid (MVA) pathway and methylerythritol phosphate (MEP) pathway, respectively (Additional file 2). Except an up-regulated gene encoding 3-hydroxy-3-methyl-glutaryl-CoA synthase (HMG-CoA synthase, HMGS), three genes encoding HMG-CoA reductase (HMGR), mevalonate kinase (MVK) and phosphomevalonate kinase (PMK) were remarkably down-regulated in the MVA pathway (Figure 6). HMGS catalyzes the condensation of Acetyl-CoA with acetoacetyl-CoA into HMG-CoA and HMGR is a rate-controlling enzyme that catalyzes the reduction of HMG-CoA to mevalonic acid (MVA). The resulting MVA is phosphorylated to mevalonic acid 5-phosphate (MVAP) by mevalonate kinase (MVK). The interconversion of IPP and DMAPP is catalyzed by IPP isomerases (IPI).

In plastids, all DEGs that encode MEP-pathway enzymes were down-regulated (Figure 6), including 1-deoxy-D-xylulose 5-phosphate (DXP) synthase (DXS), DXP reductoisomerase (DXR), 4-diphosphocytidyl-2-C-methylerythritol (CDP-ME) synthetase (CMS), CDP-ME kinase (CMK), 2-C-methyl-D-erythritol 2, 4-cyclodiphosphate (MECDP) synthase (MCS) and 1-Hydroxy-2-methyl-2-(E)-butenyl-4-diphosphate (HMBDP) synthase (HDS). First step is the condensation of (hydroxyethyl) thiamin (derived from pyruvate) and the C1 aldehyde group of D-glyceraldehyde 3-phosphate (GA-3P) by DXS. The resulting DXP was converted to MEP by DXR and CMS converts MET to CDP-ME. The hydroxyl group in the C2 position of CDP-ME is further phosphorylated by CMK. Subsequently 4-diphosphocytidyl-2-C-methyl-D-erythritol 2-phosphate (CDP-ME2P) is converted to MECDP by MCS. HDS catalyzes the conversion of MECDP to HMBDP and finally HDR catalyzes the reductive dehydration of HMBPP to yield both IPP and dimethylallyl diphosphate (DMAPP).

In addition, the expression of geranyl diphosphate (GPP) synthase (GPPS) and farnesyl diphosphate (FPP) synthase (FPPS) which catalyze the formation of GPP and FPP respectively was also dramatically down-regulated (Additional file 2).

### DEGs involved in jasmonate synthesis

**Figure 7:**
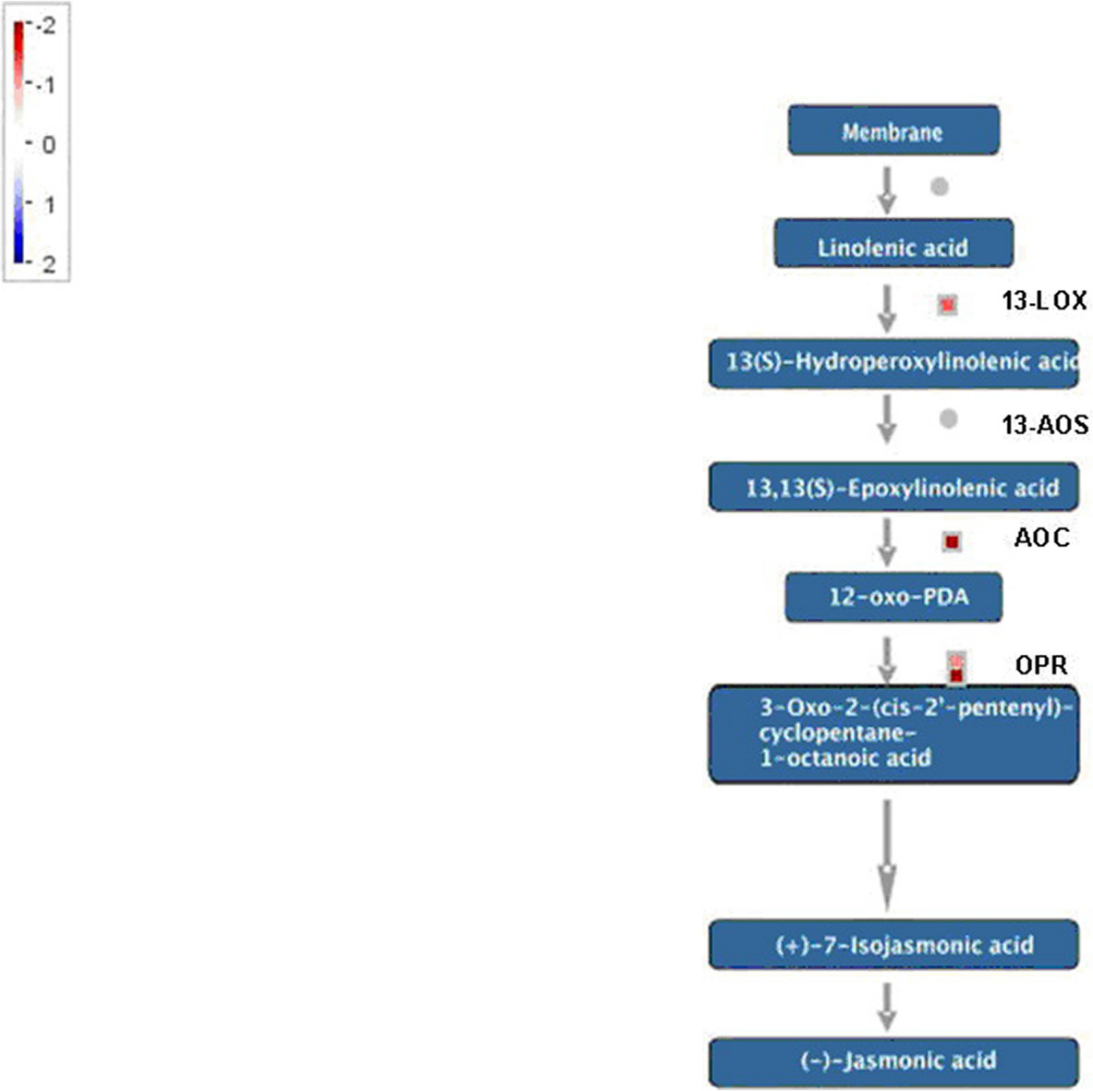
A schematic representation the differential expression of the genes coding the enzymes involved in jasmonic acid biosynthesis across the transcriptome of the TPD affected rubber trees. Color bar represents the levels of transcript abundance. The depth of the red and the blue indicates the levels of the down-regulated expression and the up-regulated expression of the genes, respectively.

In jasmonate synthesis pathway, 13-lipoxygenase (13-LOX), allene oxide cyclase (AOC) and 12-oxophytodienoic acid (OPDA) reductase (OPR) were found to be down-regulated (Figure 7; Additional file 3). In the first step, 13S-hydroperoxy-(9Z,11E,15)-octadecatrienoic acid (13-HPOT) was formed from α-linoleic acid (a-LeA) released from chloroplast membranes by a chloroplast-located 13-LOX. 12,13(S)-epoxy-octadecatrienoic acid (12,13(S)-EOT) is generated from 13-HPOT by AOS and further cyclized to OPDA by AOC. OPDA is translocated from chloroplasts to peroxisomes, where it is further converted into (+)-7-iso-JA by OPR and 3 beta oxidation steps.

### DEGs of transcription factors

TPD of rubber tree is generally considered as a complex physiological disorder, and genetic, developmental, histological, cytological and biochemical processes would be involved in. Transcription factors (TFs) understandably control and coordinate those diverse pathways and processes as master regulators. In our study, 415 proteins were identified as putative TFs categorized into different familes (Additional file 4) such as AP2/EREBP, ARR, bHLH, C2H2 zinc finger, CCAAT box binding factor, EIN3-like (EIL), G2-like, HB, HSF, MADS box, MYB box, NAC domain, Trihelix, WRKY domain, bZIP, etc. The highest down-regulated TFs in T compared to H were MYB61 (MYB DOMAIN PROTEIN 61) with alog_2_ ratio (T/H) of −8.371071 and PWWP domain-containing protein with a log_2_ ratio (T/H) of −6.1634774, and the top up-regulated TFs in T compared to H were LOL1 (LSD ONE LIKE 1) with a log_2_ ratio (T/H) of 6.102857 and zinc finger (FYVE type) family protein with a log_2_ ratio (T/H) of 7.7258673. But their exact functions in occurrence and maintenance of TPD remained to be elucidated.

### Confirmation of differential expression of the DEGs

**Figure 8:**
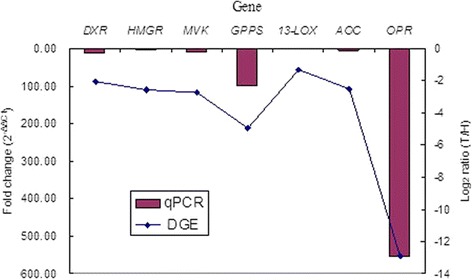
qRT-PCR confirmation of the DEGs involved in isoprenoid biosynthesis and jasmonate synthesis.Seven down-regulated genes (DXR, HMGR, MVK, GPPS, 13-LOX, AOC and OPR) in T library have been identified by qRT-PCR. The left y-axis represents the fold change by qRT-PCR and the right y-axis represents the log_2_ ratio of T/H by DGE analysis. The SDs of 2^−△△Ct^ were inferior to 0.62 and not represented.

To validate transcriptome results, differential expression of seven genes (DXR, HMGR, MVK, GPPS, 13-LOX, AOC and OPR) selected at random was verified by qRT-PCR. These eight genes were involved in isoprenoid biosynthesis and jasmonate synthesis, and their expressions were remarkably down-regulated in T library. The results obtained by qRT-PCR fitted in well with the expression pattern by transcriptome analysis (Figure 8).

## Discussion

In this study, we have first profiled the gene expression between the healthy rubber trees and the TPD-affected trees using Illumina sequencing technology. This transcriptome dataset will serve as an important public information platform for the discovery of TPD-related genes, the functional genomic investigation of TPD and the elucidation of the TPD molecular mechanism.

Previously, Venkatachalam et al. [33] and Li et al. [4] using SSH method found that stress/defense related genes represented the majority of ESTs representing genes expressed during the onset of TPD syndrome. Li et al. [4] using latex samples collected and pooled from the TPD trees at initial stage (showing a partial stoppage of latex flow) identified the differential expression of genes associated to reactive oxygen species (ROS), stress/defense, programmed cell death (PCD) and ubiquitin proteasome pathway (UPP). In present study, the majority of down-regulated GO-annotated DEGs were associated to Cellular Component (Figure 4) and up-regulated DEGs of Biological Process were predominant in T compared to H (Figure 5). This difference can mainly be attributed to different sampling strategies adopted. In out study, we used the bark samples (complete stoppage of latex flow but alive) of rubber trees for *de novo* sequencing and transcriptome profiling. The reason for this is not clear, but it is possibly that there were fewer laticifer differentiation/regeneration and more active biological processes related to the tree growth occurred in TPD-affected bark showing complete dryness, although the biosynthesis of latex was turned off. In fact, a more vigorous growth and more trunk girth increment are often observed in rubber trees after tapping cut becomes entirely dry.

Biosynthesis of natural rubber, poly(cis-1,4-isoprene), is from IPP units, and in higher plants the monomeric subunit of natural rubber IPP is formed by the MVA pathway and the MEP pathway [53,54]. Moreover, initiation of rubber biosynthesis needs a priming allylic diphosphate and FPP is the most likely initiator molecule in *H. brasiliensis* [55]. The production of the precursors of rubber biosynthesis through he MVA pathway and the MEP pathway is precisely regulated [56]. The genes of these two pathways were cloned and characterized in natural rubber tree (*H. brasiliensis*) [57,58]. HMGR, the key regulatory step for IPP biosynthesis through the MVA pathway, has been extensively studied [59,60].

In TPD affected rubber trees, latex production is severely decreased and can be completely shut down in the ultimate stage [29]. Krishnakumar et al. [61,62] suggested that the altered energy metabolism in TPD trees affected the ability of *Hevea* trees to synthesize rubber (cis-poly isoprene) and oxidative damage of laticiferous vessels may be the cause for the complete shut down of the rubber biosynthetic machinery. Chrestin et al. [63] and Chrestin [12] suggested over exploitation of *Hevea*, and in particular overstimulation with Ethrel can lead to TPD and an imbalance between the toxic peroxidative activities and the scavenging activities within the latex vessels should be responsible for the disorganization of the organelle membrane structure. Siswanto [64] concluded that the degradation of membrane lutoids by toxic forms of oxygen may be the major cause of TPD. Cloning of a Myb transcription factor HbMyb1 [3] and a TOM20 like protein gene [31] implicated that PCD in bark cells and/or alteration of mitochondrial metabolism possibly play a role in TPD occurrence. But there were some reports that sometimes trees went dry abruptly even the occurrence of TPD in trees which were not opened for tapping [65]. We first found that the expression of most genes related to the latex biosynthesis was severely inhibited and it probably the direct cause of the TPD. It is suspected that the latex production dramatically decreased or even completely ceased in TPD affected trees due to inadequate supply or depletion of their immediate substrates (IPPs and FPPs).

The expressions of the majority of genes involved in JA biosynthesis including 13-LOX, AOC and OPR were found to be down-regulated. It is noteworthy that the suppression of OPR was the greatest (with a log2 Ratio (T/H) of −12.915768) and it suggested that the OPR might be a key enzyme for JA biosynthesis pathway. As we know, the first half of the JA biosynthesis from α-LeA to OPDA occurs in a plastid, and after the transportation of OPDA into a peroxisome, the second half taking place in the peroxisome is begun with the conversion of OPDA to cyclopentanone catalyzed by OPR [66].

Interestingly, *HMGR1* in the MVA pathway was concurrently suppressed. The mevalonate is generated from HMG-CoA by HMGR and is further converted to isoprenoid compounds as well as natural rubber. Although there are genes (*HMGR1*, *HMGR2*, and *HMGR3*) encoding HMGR in *H. brasiliensis*, but only *HMGR1* is likely to be involved in rubber biosynthesis [67]. The facts that the expression of rate-limiting *HMGR1* in the MVA pathway was regulated by methyl jasmonate [68–70] suggested that the suppression of latex production is possibly achieved by decrease in JA biosynthesis. Moreover, linolenic acid (a precursor of JA biosynthesis) was shown to induce the laticifer differentiation of *H. brasiliensis* [71,72]. In some cases, JA can induce production of ROS and alterations in mitochondrial dynamics and is involved in local PCD [73,74]. Therefore, we speculate that JA may play a central role in latex production as well as in the occurrence and maintainace of TPD.

Many study showed that over-stimulation with ethphon and employing intensive tapping systems increase the incidence of dryness [12–16]. But the cause that leads to TPD seems to vary under different situations. More generally, the TPD syndrome is found to be due to a cumulative effect of many factors like over-tapping, over dosage of stimulation and sub-optimal agro-climatic conditions [75]. But there were reports that, sometimes trees went dry abruptly, and in few cases the similar dry syndrome occurred in trees which were not even opened for tapping [65].

In future, we need to research the influence of those factors on JA biosynthesis and latex biosynthesis. Especially, in the case of TPD resulted from intensive exploitation and over-stimulation with ethephon, the study should be conducted to show that JA biosynthesis is suppressed and further resulted in the decline of the latex biosynthesis after intensive exploitation and over-stimulation with ethphon. As for the phenomena that the incidence of TPD varies with varieties and the occurrence of TPD in trees which were not opened for tapping [65], it seems explicable since in some rubber clones and trees, the expression levels of those genes are possibly genetically low.

## Conclusions

Our findings shed a new light on the nature of, and even the cause of TPD. In completely dried panel trees, the expression of genes associated with the latex biosynthesis was significantly inhibited and it is possibly resulted from the reduction of the signaling molecule JA biosynthesis. In addition, the transcriptomes in this study provide valuable information for further identification of genes related with TPD.

## Materials and methods

### Plant material

The high yielding clone PR107 planted at the experimental farm of Chinese Academy of Tropical Agricultural Sciences in 2000 was selected for the present experiment. Since 2008, the rubber trees have been regularly tapped with a tapping system (S/2 d/4, i.e. half spiral cut tapped at the fourth daily frequency) plus application of 1.5% ethephon two days before tapping (once every three tappings). The bark tissues were respectively collected from nine healthy rubber trees and nine TPD-affected ones showing complete dryness. The samples were washed with diethyl pyrocarbonate treated water to remove the latex and immediately frozen in liquid nitrogen and shipped on dry ice to BGI Life Tech Co., Ltd (Shenzhen, China) for Illumina sequencing.

### RNA extraction, cDNA library construction and de novo sequencing

Bark RNA was extracted using the TRIzol® Reagent (Invitrogen) according to the manufacturer's instructions. RNA samples were detected for quantification and quality by a Nanodrop ND-1000 spectrophotometer (Thermo Scientific) and a 2100 Bioanalyzer (Agilent Technologies). Two cDNA libraries, H (healthy rubber trees) and T (TPD-affected trees), were prepared using the mRNA-Seq 8 sample prep Kit (Illumina) according to the manufacturer's instructions. Poly(A) mRNA was first isolated from 20 μg of total RNA using magnetic beads containing poly-T molecules. Then, the purified samples were fragmented into small pieces using divalent cations at 94 °C for 5 minutes and reverse transcribed into cDNAs with the SuperScript double-stranded cDNA synthesis kit (Invitrogen, CA). Then, end repair and adenylation of 3' ends were conducted and purified using the QIAquick PCR Purification Kit (QIAGEN). After that, Illumina paired end adapters were ligated to the resulting cDNA fragments. Finally, each cDNA library with an insert size of 200 bp was generated. After quality checking by an Agilent Technologies 2100 Bioanalyzer, the libraries were deep sequenced using Illumina HiSeqTM 2000 (Illumina Inc., San Diego, CA, USA).

### *De novo* assembly and gene annotation

Raw reads produced from sequencing machines were filtered by the Illumina pipeline and the dirty raw reads with adaptors and unknown nucleotides larger than 5%, and low quality reads (the rate of reads which quality value ≤ 10 is more than 20%) were removed. Transcriptome *de novo* assembly was then carried out with short reads assembling program – Trinity (release 20130225 ) (http://trinityrnaseq.github.io/) [76,77]. First, Unigenes, the result sequences of trinity, from each sample's assembly were taken into further process of sequence splicing and redundancy removing to acquire non-redundant Unigenes as long as possible. Second, the Unigenes were divided into two classes via gene family clustering: clusters (CL, there were several Unigenes which similarity between them was more than 70%) and singletons (Unigene). In the final step, Blast X alignment (an E-value < 0.00001) between the Unigenes and protein databases like NT (non-redundant NCBI nucleotide database), NCBI's non-redundant protein databases (NR), SwissProt, Kyoto Encyclopedia of Genes and Genomes (KEGG) and COG (Cluster of Orthologous Groups) was performed and the best aligning results were used to decide sequence direction of the Unigenes and assign functional annotations to the Unigenes.

With the NR annotation, the Blast2GO program [78] was used to generate the Gene Ontology (GO) annotation for the Unigenes and thereafter the WEGO software [79]) to do the GO functional classification for the Unigenes for understanding the distribution of gene functions of the species from the macro level. With the help of KEGG database [80], unigene metabolic pathway analysis was carried out to further study genes’ biological complex behaviors and get pathway annotation for the Unigenes.

### Unigene expression difference analysis

FPKM method (Fragments Per kb per Million reads) was applied to calculate the expression of the Unigenes [81]. Therefore the calculated gene expression could be directly used for comparing the difference of gene expression between the two samples. Based on the method by Audic and Claverie [82], a rigorous algorithm to identify differential transcript accumulation between the two samples was developed for screening the differentially expressed genes (DEGs). The FDR (false discovery rate) was used to determine the threshold of the P-value in multiple tests and analyse for calculating the expression between two samples [83]. In our analysis, we chose those DEGs with FDR ≤0.001 and ratio larger than 2 to conduct GO functional analysis and KEGG Pathway analysis. We using the MapMan tool view annotated processes of interest or metabolic pathways by groups of rubber tree transcripts. In order to further understand genes’ biological functions, pathway enrichment analysis was conducted to identify significantly enriched metabolic pathways or signal transduction pathways in DEGs comparing with the whole genome background [84].

### Validation of gene expression by qRT-PCR

Seven cDNAs (lowly expressed in the T library) encoding DXR (1-deoxy-D-xylulose 5-phosphate reductoisomerase), HMGR (3-hydroxy-3-methyl-glutaryl-CoA reductase), MVK (Mevalonate kinase), GPPS (geranyl diphosphate synthase), 13-LOX (13-lipoxygenase), AOC (Allene oxide cyclase) and OPR (12-oxophytodienoic acid reductase) with potential roles in rubber biosynthesis and its regulation were chosen for validation using qRT-PCR. Total RNA was respectively extracted from the equal amount of bark tissues of three H healthy rubber trees and three TPD-affected ones showing complete dryness as described by Venkatachalam et al. [31] for qRT-PCR. The first strand cDNA was converted from 2.5 μg of total RNA through a RevertAidTM Premium first strand cDNA synthesis kit (Fermentas). The standard curve for each target gene was obtained by qRT-PCR with series cDNA dilutions of cDNA. The reaction mixture (20 μl) for qRT-PCR comprised of 10 μl SYBR Premix Ex TaqII, 6.8 μl EASY Dilution, 0.6 μl 10 μM Forward primer and 0.6 μl 10 μM Reverse primer. The PCR reactions were performed on an CFX96TM Real-Time PCR Detection System (Bio-Rad) with the following program: 95 °C for 30 s, followed by 40 cycles of 95 °C for 10 s, and then annealing at 65 °C-95 °C for 30 s. The reactions of three independent biological replicates were performed for each sample with 18S rRNA gene as an internal reference and the relative expressions of the genes were calculated using the 2^−△△Ct^ method. The forward and reverse primers used in this study are listed in Table 1.

**Table 1:** The forward and reverse primers used in validation experiment of gene expression by qRT-PCR.

**Genes**	**Directions**	**Sequences**	**Length (bp)**	**Annealing Temperature (°C)**
*DXR*	Forward	CATTGGGACTCAGACATTGGACATC	215	63
	Reverse	CTTGCTCCCCAGGAATAATCTCAG		
*HMGR1*	Forward	GGCTGTGGATGATGATGAGGAGGAG	135	63
	Reverse	GGAGAAGAAACAACTGCCCGTGAC		
*MVK*	Forward	ACTTAGGTGCTCCTTCCTCTTCAAC	182	64
	Reverse	GACAACTACGGTGGCAGGCTTA		
*GPPS2*	Forward	GGCAAGCGTGTCCGTCCAGTTTT	299	64
	Reverse	TCAGCAATGGCTCTAACCACTCG		
*13-LOX3*	Forward	ATGGAGAAAAGGGAAGAGTTGATAG	248	63
	Reverse	ATAAGGGAAATGCCAATAACAGTC		
*AOC4*	Forward	AGGTGATAGATACGAAGCAACATACAG	307	64
	Reverse	AGGAATAGTCCCAGAAGACAAAGTG		
*12-OPR*	Forward	CTCACCCGAGAAGTCATACCAAAT	278	63
	Reverse	CCAACAGTGCCCACAGCCAT		
18 s rRNA	Forward	TCTCAACCATAAACGATGCCGACC	118	63
	Reverse	TTCAGCCTTGCGACCATACTCCC		

## Additional files

Additional file 1:
**List of Gene lengths, Nr annotations, Nt annotations,** **Swiss-Prot** **annotations, COG function descriptions, KEGG genes, GO biological processes and GO molecular functions in H and T Transcriptomes.**
Additional file 2:
**DEGs involved in the isoprenoid metabolism.**
Additional file 3:
**DEGs involved in the jasmonate biosynthesis pathway.**
Additional file 4:
**DEGs of transcription factors.**

## Abbreviations

ACS: Acetyl-CoA synthetase
AOC: Allene oxide cyclase
13-AOS: 13-allene oxide synthase
CMS: 4-Diphosphocytidyl-2-C-methyl-D-erythritol synthase
CDP-ME: synthase
CMK: CDP-ME kinase
COG: Cluster of Orthologous Groups
DDRT-PCR: mRNA differential display reverse transcriptase polymerase chain reaction
DEGs: differentially expressed genes
DXS: 1-deoxy-D-xylulose 5-phosphate synthase, DXP, synthase
DXR: DXP reductoisomerase
FPKM or RPKM: Fragments Per kb per Million reads
FPP: farnesyl diphosphate
FPPS: FPP synthase
GO: Gene Ontology
GPP: geranyl diphosphate
GPPS: GPP synthase
H: healthy
HDS: 1-Hydroxy-2-methyl-2-(E)-butenyl-4-diphosphate synthase
HMBDP: synthase
HDR: MMBDP reductase
HMGS: 3-hydroxy-3-methyl-glutaryl-CoA synthase
HMG-CoA: synthase
HMGR: HMG-CoA reductase
IPI: isopentenyl pyrophosphate isomerase
IPP: isomerase
KEGG: SwissProt Kyoto Encyclopedia of Genes and Genomes
13-LOX: 13-lipoxygenase
MCS: 2-C-methyl-D-erythritol 2, 4-cyclodiphosphate synthase
MECDP: synthase
MEP: methylerythritol phosphate
MVA: mevalonic acid
MVK: Mevalonate kinase: NR, non-redundant protein databases
OPR: 12-oxophytodienoic acid reductase
OPDA: reductase
PCD: programmed cell death
PCR: Polymerase chain reaction
PMK: phosphomevalonate kinase
PMD: Pyrophosphomevalonat decarboxylase or diphosphomevalonate decarboxylase
qRT-PCR: Real-time fluorescence quantitative PCR
RNA-Seq: RNA sequencing
ROS: reactive oxygen species
SSH: suppression subtractive hybridization
TFs: Transcription factors
TPD: Tapping panel dryness
T: TPD-affected
UPP: ubiquitin proteasome pathway.

## References

[1] Priyadarshan PM, Gonçalves PS, Omokhafe KO, Jain SM, Priyadarshan PM (2009). Breeding *Hevea* rubber. In *Breeding plantation tree crops: tropical species*.

[2] Mooibroek H, Cornish K (2000). Alternative sources of natural rubber. Appl Microbiol Biotechnol.

[3] Chen S-C, Peng S-Q, Huang G-X, Wu K, Fu X, Chen Z (2002). Association of decreased expression of a *Myb* transcription factor with the TPD (tapping panel dryness) syndrome in *Hevea brasiliensis*. Plant Mol Biol.

[4] Li D-J, Deng Z, Chen C-L, Xia Z-H, Wu M, He P (2010). Identification and characterization of genes associated with tapping panel dryness from *Hevea brasiliensis* latex using suppression subtractive hybridization. BMC Plant Biol.

[5] 5.Taysum HH: Yield increase by the treatment of *Hevea brasiliensis* with antibiotics. *Proceedings of the RRIM Planters' Conference*: 19–21 July 1960; Kuala Lumpur 1960:132–138.

[6] Zheng GB, Chen MR (1982). Study of the cause for brown blast disease. Chin J Trop Crops.

[7] Soyza AG (1983). The investigation of the occurring rule and distributing pattern of brown blast disease of rubber tree in Sri Lanka. J Rubber Res Inst Sri Lanka.

[8] Li ZY (1982). The relationship of brown blast of rubber trees and over exploitation. Chin J Trop Agri.

[9] Wang CZ (1988). The report about bark of TPD Hevea brasiliansis inoculating. Chin J Trop Agri.

[10] Nandris D, Chrestin H, Noirot M, Nicole M, Thouvenel JC, Geiger JP (1991). The phloem necrosis of the trunk of rubber tree in Ivory Coast. (1) Symptomatology and biochemical characteristics. Eur J For Path.

[11] Nandris D, Thouvenel JC, Nicole M, Chrestin H, Rio B, Noirot M (1991). The phloem necrosis of the trunk of the rubber tree in Ivory Coast. (2) Etiology of the disease. Eur J For Path.

[12] Chrestin H, D'Auzac J, Jacob JL, Chrestin H (1989). iochemical aspects of bark dryness induced by overstimulation of rubber trees with ethrel. In *Physiology of Rubber Tree Latex*.

[13] de Fay E, Jacob JL, D'Auzac J, Jacob JL, Chrestin H (1989). Symptomatological, histological and cytological aspects. In *Physiology of Rubber Tree Latex*.

[14] Fan SW, Yang SQ (1984). Cause of disease and hypothesis on tapping panel dryness of *Hevea brasiliensis*. Chin J of Trop Crops Res.

[15] Jacob JL, Prevot JC, Laccrotte R (1994). Tapping panel dryness in *Hevea brasiliensis*. Plantations, Recherche, Développement.

[16] Faridah Y, Siti Arija MA, Ghandimathi H (1996). Changes in some physiological latex parameters in relation to over exploitation and onset of induced tapping panel dryness. J Nat Rubber Res.

[17] Tupy J, Primot L (1976). Control of carbohydrate metabolism by ethylene in latex vessels in *Hevea brasiliensis* in relation to rubber production. Biol Plant.

[18] Pakianathan SW, Samsidar H, Sivakumaran S, Gomez JB (1982). Physiological and anatomical investigation on long term ethephon-stimulated trees. J Rubber Res Inst Malaysia.

[19] Sivakumaran S, Pakianathan S-W, Abraham PD (1984). Continuous yield stimulation. Plausible cause of yield decline. J Rubber Res Inst Malaysia.

[20] Tupy J (1969). Nucleic acid in latex and production of rubber in *Hevea brasiliensis*. J Rubber Res Inst Malaysia.

[21] Fan SW, Yang SQ (1995). Tapping panel dryness induced by excessive tapping is a local senescence phenomenon. Chin J Trop Crops Res.

[22] Zeng RZ (1997). The relation between contents of nucleic acid and tapping panel dryness in latex from *Hevea brasiliensis*. Chin J Crops.

[23] Fan XW, Yang SQ (1994). Cause of disease and hypothesis on tapping panel dryness of *Hevea brasiliensis*. China J Trop Crops Res.

[24] Xi WX, Xiao XZ (1988). Study on peroxidase isozyme and syperoxyde dismutase isozyme of TPD hevea trees. Chin J Trop Crops.

[25] Krishnakumar R, Cornish K, Jacob J (2001). Rubber biosynthesis in tapping panel dryness affected Hevea trees. J Nat Rubber Res.

[26] Chrestin H, Bangratz J, d’Auzac J, Jacob JL (1984). Role of the lutoidic tonoplast in the senescence and degeneration of the laticifers of *Hevea brasiliensis*. Z Pflanzenphysiol.

[27] Sookmark U, Pujade-Renaud V, Chrestin H, Lacotem R, Naiyanetr C, Seguin M (2002). Characterization of polypeptides accumulated in the latex cytosol of rubber trees affected by the tapping panel dryness syndrome. Plant Cell Physiol.

[28] Darussamin A, Suharyanto S, Chaidamsari T (1995). Change in the chemical composition and electrophoretic profile of latex and bark protein related to tapping panel dryness incidence in *Hevea brasiliensis*. Menara Perkebunan.

[29] Dian K, Sangare A, Diopoh JK (1995). Evidence for specific variations of protein pattern during tapping panel dryness condition development in *Hevea brasiliensis*. Plant Sci.

[30] Lacrotte R, Gidrol X, Vichitcholchai N, Pujade-Renaud V, Narangajavana J, Chrestin H (1995). Protein markers of tapping panel dryness. Plant Rech Dev.

[31] Venkatachalam P, Thulaseedharan A, Raghothama K (2009). Molecular identification and characterization of a gene associated with the onset of tapping paneldryness (TPD) syndrome in rubber tree (*Hevea brasiliensis* Muell.) by mRNA differential display. Mol Biotechnol.

[32] Venkatachalam P, Geetha N, Priya P, Thulaseedharan A (2010). Identification of a differentially expressed thymidine kinase gene related to tapping panel dryness syndrome in the rubber tree (*Hevea brasiliensis* Muell.Arg.) by random amplified polymorphic DNA screening. INT J Plant Biol.

[33] Venkatachalam P, Thulaseedharan A, Raghothama K (2007). Identification of expression profiles of tapping panel dryness (TPD) associated genes from the latex of rubber tree (*Hevea brasiliensis* Muell. Arg.). Planta.

[34] Qin B, Liu X-H, Deng Z, Li D-J (2012). Identification of genes associated with tapping panel dryness in *Hevea brasiliensis* using oligonucleotide microarrays. Chin J Trop Crops.

[35] Schuster SC (2008). Next-generation sequencing transforms today’s biology. Nat Methods.

[36] Rosenkranz R, Borodina T, Lehrach H, Himmelbauer H (2008). Characterizing the mouse ES cell transcriptome with Illumina sequencing. Genomics.

[37] Ansorge WJ (2009). Next-generation DNA sequencing techniques. N Biotechnol.

[38] Wang Z, Gerstein M, Snyder M (2009). RNA-Seq: a revolutionary tool for transcriptomics. Nat Rev Genet.

[39] Metzker ML (2010). Sequencing technologies-the next generation. Nat Rev Genet.

[40] Ozsolak F, Milos PM (2011). RNA sequencing: advances, challenges and opportunities. Nat Rev Genet.

[41] Van Verk MC, Hickman R, Pieterse CMJ, Van Wees SCM (2013). RNA-Seq: revelation of the messengers. Trends Plant Sci.

[42] Ekblom R, Galindo J (2011). Applications of next generation sequencing in molecular ecology of non-model organisms. Heredity (Edinb).

[43] Ariyurek Y, Thygesen HH, Vreugdenhil E, Vossen RH, de Menezes RX, Boer JM (2008). Deep sequencing-based expression analysis shows major advances in robustness, resolution and interlab portability over five microarray platforms. Nucleic Acids Res.

[44] Xiang LX, He D, Dong WR, Zhang YW, Shao JZ (2010). Deep sequencing based transcriptome profiling analysis of bacteria-challenged lateolabrax japonicus reveals insight into the immune-relevant genes in marine fish. BMC Genomics.

[45] Tang Q, Ma XJ, Mo CM, Wilson IW, Song C, Zhao H (2011). An efficient approach to finding *Siraitia grosvenorii* triterpene biosynthetic genes by RNAseq and digital gene expression analysis. BMC Genomics.

[46] Xia Z, Xu H, Zhai J, Li D, Luo H, He C (2011). RNA-Seq analysis and de novo transcriptome assembly of *Hevea brasiliensis*. Plant Mol Biol.

[47] Pootakham W, Chanprasert J, Jomchai N, Sangsrakru D, Yoocha T, Therawattanasuk K (2011). Single nucleotide polymorphism marker development in the rubber tree, *Hevea brasiliensis* (Euphorbiaceae). Am J Bot.

[48] Triwitayakorn K, Chatkulkawin P, Kanjanawattanawong S, Sraphet S, Yoocha T, Sangsrakru D (2011). Transcriptome sequencing of *Hevea brasiliensis* for development of microsatellite markers and construction of a genetic linkage map. DNA Res.

[49] Li D, Deng Z, Qin B, Liu X, Men Z (2012). De novo assembly and characterization of bark transcriptome using Illumina sequencing and development of EST-SSR markers in rubber tree (*Hevea brasiliensis* Muell. Arg.). BMC Genomics.

[50] Chow K-S, Mat-Isa MN, Bahari A, Ghazali A-K, Alias H, Mohd-Zainuddin Z (2012). Metabolic routes affecting rubber biosynthesis in *Hevea brasiliensis* latex. J Exp Bot.

[51] Duan C, Argout X, Gébelin V, Summo M, Dufayard JF, Leclercq J (2013). Identification of the Hevea brasiliensis AP2/ERF superfamily by RNA sequencing. BMC Genomics.

[52] Rahman AY, Usharraj AO, Misra BB, Thottathil GP, Jayasekaran K, Feng Y (2013). Draft genome sequence of the rubber tree *Hevea brasiliensis*. BMC Genomics.

[53] Gronover CS, Wahler D, Prüfer D, Elnashar M (2011). Natural rubber biosynthesis and physicchemical studies on plant derived latex. In *Biotechnology of Biopolymers*.

[54] Cornish K (2001). Biochemistry of natural rubber, a vital raw material, emphasizing biosynthetic rate, molecular weight and compartmentalization, in evolutionarily divergent plant species. Nat Prod Rep.

[55] da Costa BMT, Keasling JD, McMahan CM, Cornish K (2006). Magnesium ion regulation of *in vitro* rubber biosynthesis by *Parthenium argentatum* Gray. Phytochemistry.

[56] Okada K (2011). The biosynthesis of isoprenoids and the mechanisms regulating it in plants. Biosci Biotechnol Biochem.

[57] Sando T, Takaoka C, Mukai Y, Yamashita A, Hattori M (2008). Cloning and characterization of mevalonate pathway genes in natural rubber producing plant. Hevea brasiliensis. Biosci Biotechnol Biochem.

[58] Sando T, Takeno S, Watanabe N, Okumoto H (2008). Cloning and characterization of the 2-C-methyl-D-erythritol 4-phosphate (MEP) pathway genes of a natural-rubber producing plant. Hevea brasiliensis. Biosci Biotechnol Biochem.

[59] Brown MS, Goldstein JL (1980). Multivalent feedback regulation of HMG CoA reductase, a control mechanism coordinating isoprenoid synthesis and cell growth. J Lipid Res.

[60] Chang W-C, Song H, Liu H-W, Liu P-H (2013). Current development in isoprenoid precursor biosynthesis and regulation. Curr Opin Chem Biol.

[61] Krishnakumar R, Sreelatha S, Thomas M, Gopalakrishnan J, Jacob J, Sethuraj MR (1999). Biochemical composition of soft bark tissues in *Hevea* affected by tapping panel dryness. Indian J Nat Rubber Res.

[62] Krishnakumar R, Annamalainathan K, Simon SP, Jacob J (2001). Tapping panel dryness syndrome in *Hevea* increases dark respiration but not ATP status. Indian J Nat Rubber Res.

[63] Chrestin H, Jacob JL, D’Auzac J (1985). Biochemical basis for cessation of latex flow and occurrence of physiological bark dryness. Kuala Lumpur, Malaysia: Proceedings of the International Rubber Conference.

[64] Siswanto (1994). Physiological mechanism related to latex production of *Hevea brasiliensis*. Buletin-Bioteknologi-Perkebunan.

[65] Sethuraj MR (1989). Present status of investigations in the Rubber Research Institute of India on panel dryness syndrome.

[66] Kombrink E (2012). Chemical and genetic exploration of jasmonate biosynthesis and signaling paths. Planta.

[67] Suwanmanee P, Sirinupong N, Suvachittanont W: Regulation of 3-hydroxy-3-methylglutaryl-CoA synthase and 3-hydroxy-3-methylglutaryl-CoA reductase and rubber biosynthesis of *Hevea brasiliensis* (B.H.K.) Mull. Arg. In *Isoprenoid Synthesis in Plants and Microorganisms: New Concepts and Experimental Approaches*. Edited by Bach TJ, Rohmer M. Berlin/Heidelberg/New York: Springer-Verlag; 2013:315–28.

[68] Burnett RJ, Maldonado-Mendoza IE, McKnight TD, Nessler CL (1993). Expression of a 3-hydroxy-3-methylglutaryl coenzyme a reductase gene from *Camptotheca acuminate*: Is differentially regulated by wounding and methyl Jasmonate. Plant Physiol.

[69] Mehrjerdi MZ, Bihamta M-R. Omidi M, a Naghavi M-R, Soltanloo H, Ranjbar M: Effects of exogenous methyl jasmonate and 2-isopentenyladenine on artemisinin production and gene expression in *Artemisia annua*. Turk J Bot. 2013;37:499–505.

[70] Choi D, Bostock RM, Avdiushko S, Hildebrand DF (1994). Lipid-derived signals that discriminate wound- and pathogen-responsive isoprenoid pathways in plants: methyl jasmonate and the fungal elicitor arachidonic acid induce different 3-hydroxy-3-methylglutaryl-coenzyme A reductase genes and antimicrobial isoprenoids in *Solanum tuberosum* L. Proc Natl Acad Sci U S A.

[71] Hao BZ, Wu JL (2000). Laticifer differentiation in Hevea brasiliensis: induction by exogenous jasmonic acid and linolenic acid. Ann Bot.

[72] Shi M-J, Tian W-M (2012). Effect on the induction of the secondary laticifer differentiation by the transportation of exogenous JA in *Hevea brasiliensis*. Chin J Trop Crops.

[73] Zhang L, Xing D (2008). Methyl jasmonate induces production of reactive oxygen species and alterations in mitochondrial dynamics that precede photosynthetic dysfunction and subsequent cell death. Plant Cell Physiol.

[74] Vanková R, Pessarakli M (2010). Plant hormone functions in abiotic and biotic stress responses. In *Handbook of Plant and Crop Stress*, 3rd Edition.

[75] Das G, Raj S, Pothen J, Sethuraj MR, Sinha TP, Sen-Mandi S (1998). Status of free radical and its scavenging system with stimulation in *Hevea brasiliensis*. Plant Physio Biochem.

[76] Grabherr MG, Haas BJ, Levin JZ, Thompson DA, Amit I, Adiconis X (2011). Full-length transcriptome assembly from RNA-Seq data without a reference genome. Nat Biotech.

[77] Iseli C, Jongeneel CV, Bucher P. ESTScan: a program for detecting, evaluating, and reconstructing potential coding regions in EST sequences. Proc Int Conf Intell Syst Mol Biol. 1999;138–48. 10786296

[78] Conesa A, Götz S, García-Gómez JM, Terol J, Talón M, Robles M (2005). Blast2GO: a universal tool for annotation, visualization and analysis in functional genomics research. Bioinforma.

[79] Ye J, Fang L, Zheng H, Zhang Y, Chen J, Zhang Z (2006). WEGO: a web tool for plotting GO annotations. Nucleic Acids Res.

[80] Kanehisa M, Araki M, Goto S, Hattori M, Hirakawa M, Itoh M (2008). KEGG for linking genomes to life and the environment. Nucleic Acids Res.

[81] Mortazavi A, Williams BA, McCue K, Schaeffer L, Wold B (2008). Mapping and quantifying mammalian transcriptomes by RNA-Seq. Nat Methods.

[82] Audic S, Claverie JM (1997). The significance of digital gene expression profiles. Genome Res.

[83] Benjamini Y, Yekutieli D (2001). The control of the false discovery rate in multiple testing under dependency. Ann Stat.

[84] Li X-Y, Sun H-Y, Pei J-B, Dong Y-Y, Wang F-W, Chen H (2012). De novo sequencing and comparative analysis of the blueberry transcriptome to discover putative genes related to antioxidants. Gene.

